# Use of androgens at different stages of life: climacterium: Number 1 - January 2022

**DOI:** 10.1055/s-0041-1740936

**Published:** 2022-01-29

**Authors:** Andrea Prestes Nácul, Gabriela Pravatta Rezende, Daniela Angerame Yela Gomes, Técia Maranhão, Laura Olinda Bregieiro Fernandes Costa, Fernando Marcos dos Reis, Gustavo Arantes Rosa Maciel, Lia Cruz Vaz da Costa Damásio, Ana Carolina Japur de Sá Rosa e Silva, Vinicius Medina Lopes, Maria Cândida Baracat, Gustavo Mafaldo Soares, José Maria Soares, Cristina Laguna Benetti-Pinto

**Affiliations:** 1 Unidade de Reprodução Humana, Hospital Fêmina, Grupo Hospitalar Conceição, Porto Alegre, RS, Brazil; 2 Universidade Estadual de Campinas, Campinas, SP, Brazil; 3 Universidade Estadual de Campinas, Campinas, SP, Brazil; 4 Universidade Federal do Rio Grande do Norte, Natal, RN, Brazil; 5 Universidade de Pernambuco, Recife, PE, Brazil; 6 Universidade Federal de Minas Gerais, Belo Horizonte, MG, Brazil; 7 Faculdade de Medicina, Universidade de São Paulo, São Paulo, SP, Brazil; 8 Universidade Federal do Piauí, Teresina, PI, Brazil; 9 Faculdade de Medicina de Ribeirão Preto, Ribeirão Preto, SP, Brazil; 10 Universidade de Brasília, Brasília DF, Brazil; 11 Faculdade de Medicina, Universidade de São Paulo, São Paulo, SP, Brazil; 12 Universidade Federal do Rio Grande do Norte, Natal, RN, Brazil; 13 Faculdade de Medicina, Universidade de São Paulo, São Paulo, SP, Brazil; 14 Universidade Estadual de Campinas, Campinas, SP, Brazil

## Key points

- Female sexual dysfunction (FSD) includes hypoactive sexual desire disorders (HSDD) and sexual arousal, orgasm disorders and genito pelvic pain disorder, and vaginal penetration disorders.
- Female sexual dysfunction affects around 45% of women, most of them postmenopausal.
- The genitourinary menopause syndrome (GMS) includes signs and symptoms related to atrophy of the genital tract and predisposes to vaginal and/or urinary infections, in addition to interfering with the woman's sexual performance.
- There is a decline in cognitive function in postmenopausal women, and estrogens and androgens appear to independently influence cognitive activity.
- The characterization of postmenopausal androgen deficiency and the prescription of androgen therapy is still a controversial topic.

## Recommendations

- It is not recommended to establish a diagnosis of androgen insufficiency based on low concentrations of serum androgens.
- Androgens are indicated for the treatment of FSD, although until now no specific androgen therapy is approved by the Food and Drug Administration (FDA). Data are insufficient to ensure long-term efficacy and safety.
- Patients should be counseled on the scarcity of long-term safety studies. In the short term, the most reported adverse events are greater hair growth at the application site and acne.
- Physiological doses of transdermal testosterone associated or not with estrogen therapy are effective for the treatment of HSDD in postmenopausal women, but there are no formulations available in Brazil so far.
- Testosterone gel formulated in compounding pharmacies can be considered a therapeutic option for HSDD in postmenopausal women, as it is the only form of drug treatment with natural testosterone available to date.
- It is recommended to dose testosterone before starting treatment and after three to six weeks of use in order to avoid supraphysiological levels, in addition to monitoring the appearance of potential effects of excess androgens.
- If there is no satisfactory improvement in HSDD within six months of testosterone use, treatment should be discontinued. Data on the safety of treatment after two years of use are unavailable.
- Vaginal dehydroepiandrosterone (DHEA) was recently approved by the FDA [prasterone (Intrarosa®)] for the treatment of genitourinary menopause syndrome, but it is unavailable in Brazil to date. It has shown effectiveness in the treatment of dyspareunia due to atrophy of the vaginal mucosa.
- There is no evidence to recommend the use of androgens to delay cognitive decline.
- Given the paucity of more consistent studies, treatment with androgens to improve postmenopausal bone mass is not recommended.

## Background

Female sexual disfunction encompasses hypoactive sexual desire disorder (HSDD), defined as the recurrent absence or lack of fantasies and desire to have sex, associated with marked suffering or interpersonal difficulties, not explained by another mental or physical disorder, medical condition or asexuality, and female sexual arousal disorder, currently considered a single category according to the DSM-5 (5 ^th^ edition of the American Psychiatric Association Diagnostic and Statistical Manual of Mental Disorders). 1 Dyspareunia and vaginismus, currently included in the genito pelvic pain and disorders of vaginal penetration category, are also part of the FSD. 1 Considering that most studies evaluating the use of testosterone in FSD have been conducted in women with HSDD, the new diagnostic categories have not been validated in clinical studies nor are uniformly accepted by experts in the field. 1 Thus, evidences that the female sexual function (SF) is associated with androgenic action are based, above all, on studies that observed an improvement in HSDD in postmenopausal women treated with testosterone. 2 3 4 5

## What is the evidence for the different forms of androgen therapy in the treatment of FSD?

There is a consensus that FSD is multifactorial and influenced by numerous clinical, surgical, interrelational and psychosocial conditions, including hormonal changes resulting from ovarian failure during the climacteric period. 6 7 The decline in androgen production coincides with the reduction of sexual fantasies and motivation in postmenopausal women, suggesting a correlation with dysfunctional sexual behavior. 7 Although the use of testosterone in the treatment of hypoactive sexual desire is supported by the Endocrine Society 6 and the American College of Obstetricians and Gynecologists, 8 there is no FDA-approved specific androgen therapy for the treatment of FSD to date.

### Transdermal testosterone - patches

Transdermal testosterone has been the most studied. Evidence with a high degree of recommendation has shown that the use of 150 to 300 mcg of transdermal testosterone for the treatment of HSDD in women with natural or surgical menopause, with or without estrogen therapy, improves sexual desire, sexual satisfaction and the frequency of intercourse and orgasms. 2 3 4 5 9 10 However, evidence regarding long-term safety and efficacy is limited. In most studies, the usage time was not longer than six months. The patch, the most studied transdermal form in the literature, is not available in Brazil. In addition, the FDA has disapproved of the continued use of testosterone patches for lack of long-term safety evidence.

### Transdermal testosterone - gel or creams

There are no testosterone gel preparations in suitable doses for climacteric women for the treatment of FSD approved by the FDA or regulatory agencies in other countries. Australia is the only exception, where a 1% testosterone in cream is available in doses that maintain plasma levels of testosterone in the physiological limits of pre-menopause (Androfeme® 1, 0.5 g/day), and the effects of excess androgens are rare. Testosterone gel 1% (Libigel®) was tested in the US, but showed no improvement in FSD during phase 3 of a large clinical trial and was discontinued by the FDA. 1 Testosterone approved for the treatment of male hypogonadism, including injections, subcutaneous implants and gels is strongly disapproved for use in climacteric women. As testosterone levels in women represent approximately 10% of male levels, there is a significant risk of supraphysiological doses of testosterone with adverse effects, some of which are irreversible. 6 As an alternative, 1% testosterone in high absorption cream or gel prepared in compounding pharmacies for transdermal use with systemic effect has been prescribed. The recommended dose is 0.5 g of gel or cream per day, equivalent to 5 mg of testosterone per day. It should be applied on the inner thigh, buttocks or lower abdomen, and not on the arms or trunk, avoiding the lymphatic system in the breast region. Hand washing after application is recommended to prevent transfer of the product to other people. As there is no approval by the FDA or regulatory bodies in Brazil, it is difficult to assess and prove the pharmacokinetic and pharmacodynamic properties of manipulated drugs. Thus, the plasma levels of the active substance may vary between batches of the product. 11 In addition, other variables can interfere with the absorption of manipulated preparations not securely standardized, such as the use of various active substance release vehicles (creams, gels, alcoholic medium), the body location and body surface area where the medication is applied. 11 Thus, the efficacy and adverse effects of manipulated preparations cannot be fully anticipated. Another relevant aspect is that the production and consumption of manipulated hormones are not subject to systematic pharmacovigilance and notification of adverse effects, which creates the mistaken interpretation that manipulated hormones are safer. Despite restrictions on safety, testosterone manipulated in gel or cream may be considered for the treatment of HSDD, as it is the only form of natural testosterone available for transdermal use.

Some recommendations for prescribing and monitoring the treatment with manipulated testosterone: 1 6 12

- Indication for postmenopausal women with an accurate diagnosis of HSDD without contraindications to the use of hormonal therapy associated or not with estroprogestative therapy.
- Prescribe 1% testosterone formulated in a high absorption gel (eg Pentravan) for transdermal use at a dose of 0.5 g of gel per day for three to six months. As a suggestion for prescription, testosterone 5 mg per mL in a measuring bottle containing 30 mL with a release of 1 mL per day is recommended. This dose can be individualized with a variation between 1 and 5 mg. If there is improvement, reinforce to the patient that there is no evidence of efficacy and safety in use for a period longer than 24 months.
- Dose the testosterone before starting, after three to six weeks of use and while the treatment lasts to avoid supraphysiological plasma levels, and monitor the appearance of clinical signs of hyperandrogenism, because the clinical response does not always correlate with plasma levels of testosterone. 1 12
- In the presence of a satisfactory therapeutic result, maintain the clinical and laboratory evaluation described above every three to six months.
- Discontinue treatment when no improvement in FSD is observed after six months of use.

### Subdermal testosterone implants

Subdermal testosterone implants should be avoided because of the potential for adverse effects from prolonged exposure to high doses of testosterone, especially in biodegradable implants that cannot be removed from the application site. 13 These are not available in Brazil, unless in manipulation laboratories, nor are approved by regulatory agencies. 14

### Oral testosterone

Oral testosterone (eg, methyltestosterone) is not recommended because of its high biological potency, potential risk of adverse effects and hepatotoxicity. 1 8

### Intramuscular testosterone

Intramuscular administration of testosterone is not recommended because the plasma levels are often supraphysiological and there are important side effects, some of which irreversible. 8

### Vaginal testosterone

The use of vaginal testosterone was evaluated in studies with a small sample and few weeks of follow-up without proven effectiveness and safety yet. 8 Phase 2 clinical trials have evaluated new presentations of testosterone alone or associated with other drugs, such as oral testosterone associated with buspirone or sildenafil and nasal application testosterone, although with few promising results. 1

### DHEA

The systemic use of DHEA for the treatment of HSDD in postmenopausal women has no proven efficacy. 15 In addition, the endocrinology societies do not recommend its use due to the lack of evidence of long-term safety. 16 Dehydroepiandrosterone replacement is recommended for women with adrenal insufficiency with FSD and low plasma levels of DHEA, with starting doses between 25 and 50 mg per day for a period of three to six months and dose adjustments according to circulating levels of DHEA and clinical symptoms. In the absence of a satisfactory therapeutic result or the presence of adverse effects, therapy should be suspended. 6 Dehydroepiandrosterone (25 to 50 mg per day) is marketed in the US as a dietary supplement, even though high doses can induce androgenic effects such as hirsutism and acne. As supplements typically receive minimal regulatory surveillance, available presentations may vary in quality, purity, and concentrations. 17

## What are the side effects associated with the use of transdermal testosterone at a physiological dose?

The main adverse effects associated with the use of transdermal testosterone in postmenopausal women at physiological doses, are mild acne and hirsutism and rarely alopecia, voice thickening or clitoromegaly. 12 At physiological doses, it has not been associated with significant effects in the lipid profile and the levels of blood pressure, blood glucose and glycated hemoglobin. A trend towards a higher risk for deep vein thrombosis has been observed, although the effect of estrogen therapy, usually associated with hormonal therapy regimens, cannot be excluded. Data to assess the effects of testosterone therapy on the risk of coronary heart disease are insufficient. 18 Endometrial abnormalities were not found after 12 months of transdermal testosterone patch use. In patients who bled during treatment, histopathology revealed atrophic endometrium. 9 The transdermal testosterone patch at physiological doses for a period not exceeding six months was not associated with higher mammographic breast density or risk of breast cancer. Current data are insufficient to ensure the absence of long-term risk. The use of testosterone in women with breast cancer with hormone receptors is not recommended. 18

## Is there an indication for the use of androgens in GMS?

Estrogen replacement was the main form of treatment and considered the gold standard for treating GMS. 19 20 Estrogen and androgen receptors and androgen-dependent proteins are distributed in the female genitourinary tract and exert a trophic effect. 20 The progressive reduction in androgen production is an additional factor in the onset of signs and symptoms of GMS. 15 16 20

The use of intravaginal testosterone at a dose of 300 mcg of testosterone daily for four weeks was effective in restoring the vaginal epithelium, reducing symptoms of vaginal atrophy and dyspareunia and improving libido, without increasing serum levels nor clinical signs of hyperandrogenism. 21 22 However, to date, there is no proven safety and efficacy for the recommendation of intravaginal testosterone in postmenopausal GMS. Dehydroepiandrosterone (6.5 mg) in the form of vaginal eggs was recently approved by the FDA [prasterone (Intrarosa®)] for the treatment of GMS, although it is still unavailable in Brazil. 1 Although the DHEA is converted into estrogen and testosterone by the vaginal cells, 17 plasma levels of estradiol, DHEA, testosterone or androstenedione possibly do not change after vaginal administration of DHEA, and laboratory monitoring is not necessary. 23 The effects of vaginal DHEA application have not been studied in women with a history of breast cancer, nor in other estrogen-dependent neoplasms. It is not indicated for the treatment of HSDD or other domains of sexual dysfunctions. 24 In conclusion, androgens seem to independently contribute to the maintenance of the structure and function of the genitourinary tissue. The effects of androgens on cell proliferation, collagen turnover, higher perfusion and neurotransmitter synthesis may complement the estrogenic action. 16

## Is androgen therapy indicated to improve cognitive function?

Postmenopausal women using injectable testosterone and estrogen showed improvement in verbal memory, suggesting that estrogen and testosterone independent effects would be neuroprotective. 25 In addition to its neuroprotective action, a positive endothelial action of testosterone has also been demonstrated, promoting arterial vasodilation. 26 However, in another clinical trial using oral testosterone undecanoate, a negative response for immediate verbal memory was obtained. 27 The use of estrogen plus methyltestosterone resulted in better memory building performance compared to the use of estrogen alone. 28 However, the divergent results between studies do not allow definitive conclusions. Dehydroepiandrosterone sulfate (SDHEA) has also shown neuroprotective effects. Women aged 21 to 77 years who had higher serum levels of SDHEA demonstrated better performance in executive functions, especially those with more than 12 years of education and high scores on simple concentration tests as well as on memory tests. 29 However, other studies with SDHEA have not shown positive results. 30 Assessments of cognitive function with androgen therapy in postmenopausal women have inconsistent results, usually in small population samples for a short period of time and using doses that are expressed in supraphysiological androgenic plasma levels. Thus, there is insufficient evidence to support the use of androgens in order to delay the decline in postmenopausal cognitive action.

## Is there evidence to indicate androgen therapy in this period of life, considering its effects on the musculoskeletal system?

Estrogen deficiency represents an important risk factor for osteoporosis. Previous studies show that androgens play an enhancing role in the formation of bone mass. 31 However, the role of testosterone in preserving bone mass in postmenopausal women is not fully recognized. A study of late postmenopausal women showed a correlation between circulating androgens and trabecular and cortical bone mineral density. 32 Investigations on the effect of androgens on the bone system are not frequent and when available, include the use of small hormonal doses for a short time. Few studies have evaluated the influence of androgens on the frequency of postmenopausal fractures. In premature ovarian insufficiency, the inclusion of androgens in estrogen replacement therapy did not show a significant increase in bone mass compared to estrogen therapy alone. 33 In surgical menopause, the use of methyltestosterone 2.5 mg daily associated with estrogens showed a significant increase in bone mass in the hip and lumbar spine. 34 The androgenic effects (T and DHEA) on the musculoskeletal system are undefined, because studies are scarce and have methodological limitations. Thus, the available studies are insufficient to indicate androgen therapy in postmenopausal musculoskeletal disorders.

## Final considerations

The use of androgens in postmenopausal women is limited and the evidence supports their use for the treatment of hypoactive desire. Evidence to support other indications is lacking.

National Specialty Commission on Gynecology Endocrinology of the Brazilian Federation of Gynecology and Obstetrics Associations (FEBRASGO)

President:

Cristina Laguna Benetti Pinto

Vice-President:

Ana Carolina Japur de Sá Rosa e Silva

Secretary:

José Maria Soares Júnior

Members:

Andrea Prestes Nácul

Daniela Angerame Yela

Fernando Marcos dos Reis

Gabriela Pravatta Rezende

Gustavo Arantes Rosa Maciel

Gustavo Mafaldo Soares

Laura Olinda Rezende Bregieiro Costa

Lia Cruz Vaz da Costa Damásio

Maria Candida Pinheiro Baracat Rezende

Sebastião Freitas de Medeiros

Tecia Maria de Oliveira Maranhão

Vinicius Medina Lopes
